# Prognosis and Treatment Effectiveness of Austrian Syndrome: A Case Report and Systematic Review

**DOI:** 10.7759/cureus.74971

**Published:** 2024-12-02

**Authors:** Takahiko Fukui, Shinsuke Muraoka, Takumi Asai, Toshihisa Nisizawa, Yoshio Araki, Ryuta Saito

**Affiliations:** 1 Neurosurgery, Kariya Toyota General Hospital, Kariya, JPN; 2 Neurosurgery, Japanese Red Cross Nagoya Daini Hospital, Nagoya, JPN; 3 Neurosurgery, Nagoya University Hospital, Nagoya, JPN

**Keywords:** austrian syndrome, cerebral infarction, endocarditis, meningitis, pneumonia, streptococcus pneumoniae

## Abstract

Austrian syndrome is a rare triad of meningitis, pneumonia, and endocarditis caused by *Streptococcus pneumoniae*. It is associated with high morbidity and mortality rates. Most reports describe pneumonia as the initial illness, followed by multi-organ involvement. Here, we present a case of Austrian syndrome initially diagnosed with cerebral infarction. The patient died due to the rapid progression of symptoms. There are few reports of cerebral infarction as an initial symptom; therefore, the relationship between the presence of cerebral infarction and its prognosis is not well known. We did a systematic review of the literature to investigate articles from 1980 to 2022. Austrian syndrome initiated by cerebral infarction was not associated with mortality in these articles. Valve vegetation results in congestive heart failure and this may be related to patient mortality. Therefore, valve replacement surgery in an appropriate time period is critical for patient mortality regardless of the presence or absence of cerebral infarction.

## Introduction

*Streptococcus pneumoniae* is the most common bacteria in patients with community- and hospital-acquired pneumonia and bacterial meningitis [1]. Austrian syndrome, also known as Osler's triad, is a rare pathology of meningitis, pneumonia, and endocarditis caused by *S. pneumoniae* [2]. Most reports describe pneumonia as the initial illness followed by multi-organ involvement [2,3].

*S. pneumoniae* endocarditis confers a high mortality risk without surgical treatment (> 60%), with the native aortic valve being the most frequent site of vegetation. Early diagnosis of heart failure due to valvular rupture is essential for a better prognosis [2]. Cerebral infarction has been reported to be associated with endocarditis. As a vascular complication, embolism of cerebral arteries occurs in about 30% of the cases [4], and may be the initial symptom to be noted in the diagnosis of infective endocarditis (IE). Cerebral infarction occurs with valvular disease of the left heart system [5]. In a review by Novy et al., they noted that about 80% of the patients with IE have some abnormality on the brain magnetic resonance imaging (MRI), and cerebral infarction is seen in 50% of the cases [6].

There are few existing reports of cerebral infarction as an initial symptom of Austrian Syndrome; therefore, the relationship between the presence of cerebral infarction and its prognosis is not well known. Here, we present a case of Austrian syndrome in a patient who was initially diagnosed with cerebral infarction. We then summarize the literature on Austrian syndrome from the viewpoint of cerebral infarction and its prognosis.

## Case presentation

**Figure 1:**
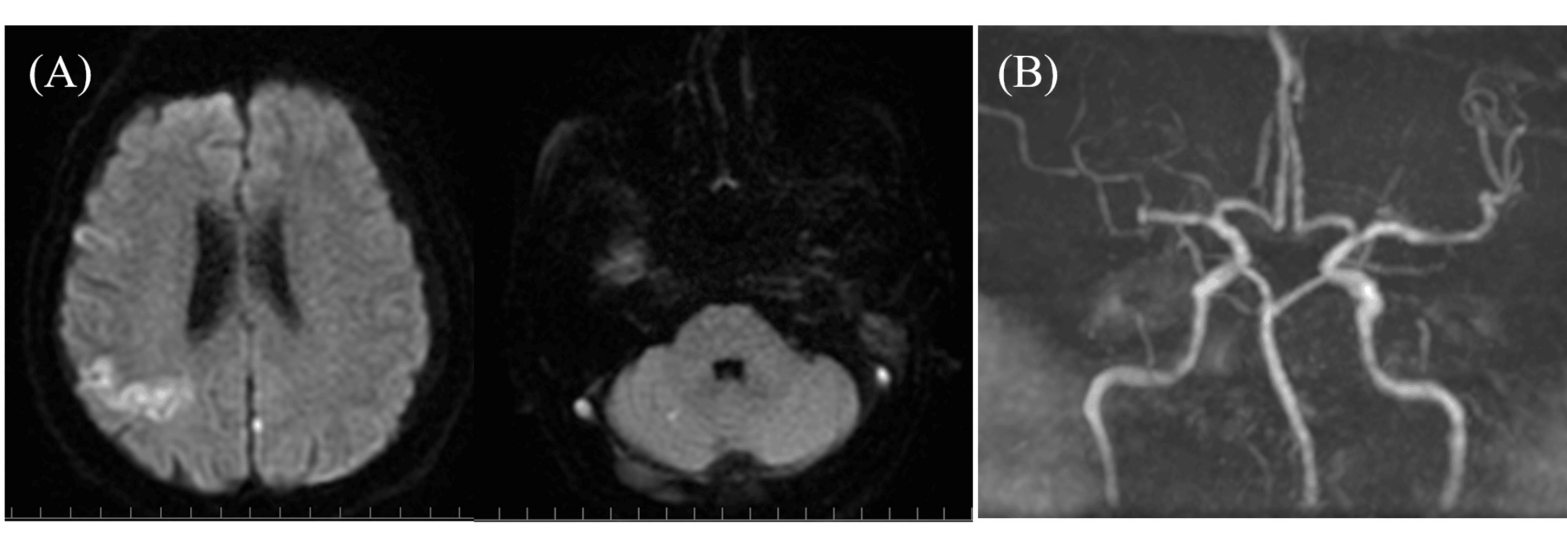
Head magnetic resonance image on admission: (A) Axial diffusion-weighted image shows multiple cerebral infarctions; (B) Magnetic resonance angiography shows no significant major arterial stenosis.

A 68-year-old male patient was admitted to our emergency department because of impaired consciousness. The patient was single, and his social and medical histories were not well known. Physical examination revealed a body temperature of 37.4 °C, blood pressure of 136/88 mmHg, heart rate of 75 beats/minute, oxygen saturation of 94% on room air, a level of consciousness of Glasgow Coma Scale (GCS) 12 (E4V3M5), left hemispatial neglect, and a National Institute of Health Stroke Scale (NIHSS) score of 10. Head MRI showed multiple acute small infarcts in the bilateral cerebrum and cerebellum but magnetic resonance angiography (MRA) showed no significant major arterial stenosis (Figure 1).

Blood tests showed an increased inflammatory response (white blood cell count: 17,100/μL and C-reactive protein: 36 mg/dL). Chest radiography revealed a mild consolidation in the right superior lung lobe. The patient was diagnosed with cerebral infarction and pneumonia.

Antiplatelet and antibiotic therapy with meropenem (2 g x 3/day) were initiated. The patient began to develop progressive fevers up to 40 ℃. About six hours after admission, the patient suddenly experienced cardiopulmonary arrest. Immediately after one intravenous administration of epinephrine, spontaneous circulation was restored to a deep comatic state. Spontaneous breathing was weak, and the patient was intubated and placed on a ventilator.

**Figure 2:**
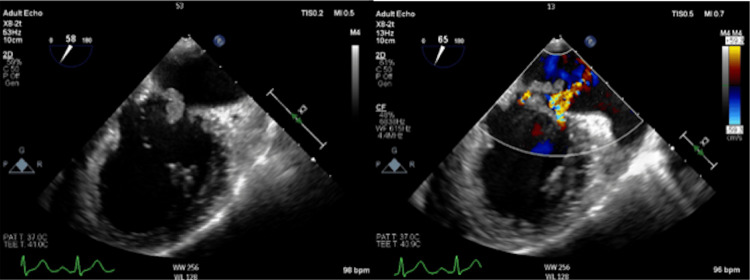
Transesophageal echocardiogram (long-axis view) reveals vegetation on the mitral valve (8–20 mm) and severe mitral regurgitation.

Transesophageal echocardiography revealed a large mitral valve verruca (Figure 2). Antibiotic therapy was escalated to meropenem (2 g x 3/day) and vancomycin (1 g x 2/day). Blood cultures revealed *S. pneumoniae*. A spinal fluid examination revealed bacterial meningitis with a glucose level of 2 mg/dL and a cell count of 1,006/μL. Although severe mitral valve regurgitation was observed, the consciousness did not improve; therefore, surgery was not indicated. The patient died two weeks after admission.

## Discussion

We discussed a case of a rapidly progressive clinical course of Austrian syndrome that initially presented as ischemic stroke.

Systematic review

Methods

A systematic review of the literature was done, following Preferred Reporting Items for Systematic Reviews and Meta-Analyses (PRISMA) 2020 guidelines. The PubMed database was searched for relevant articles published between 1980 and 2022. Initially, reports were searched using the term "Austrian syndrome". The reference lists of the articles were manually checked. Only articles written in English were included in this study. After the initial search, the titles and abstracts were checked. Finally, papers that met the inclusion criteria were subjected to full-text review by two reviewers (SM and TF). If the full-text article could not be obtained or the types of therapy and prognosis were not included, the articles were excluded.

The following data were extracted from each report: first author’s name, date of publication, age at diagnosis, sex, days until diagnosis, comorbidities, head MRI, time to diagnosis of cerebral infarction, Glasgow Coma Scale score on admission, cardiac complications, valve vegetation site, other complications, initial antibiotic therapy, valve replacement surgery, and outcome.

Statistical analyses were performed using IBM SPSS Statistics for Windows, Version 20.0 (Released 2011; IBM Corp, Armonk, New York, United States). We evaluated the relationship between cerebral infarction, initial diagnosis, and prognosis. Fisher’s exact test or χ2 test was used to compare the proportions of categorical variables between the groups. Binary logistic regression analysis was performed to calculate the odds ratio and corresponding confidence interval. Statistical significance was set at p < 0.05.

Results

**Figure 3:**
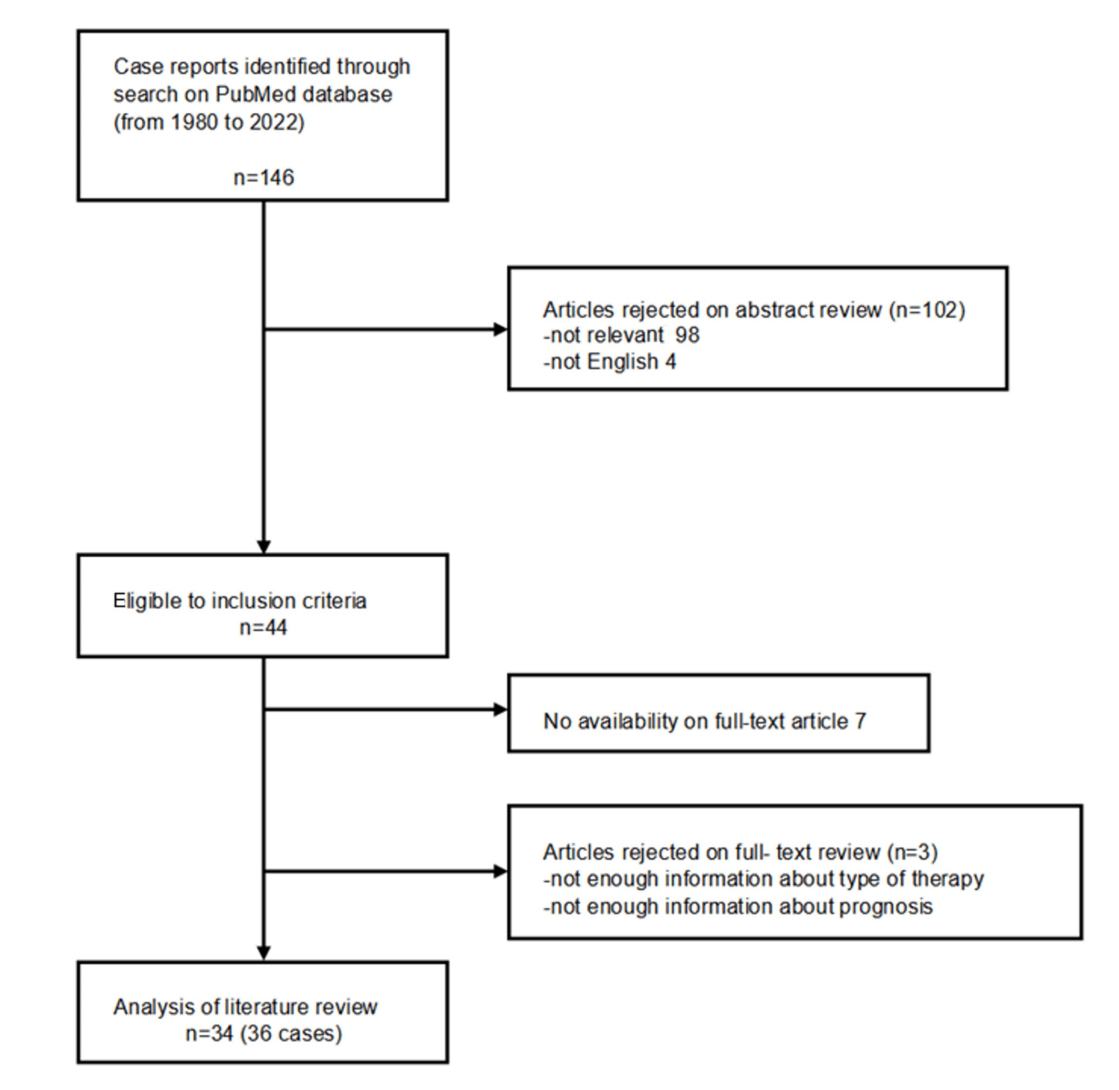
PRISMA flowchart showing study selection PRISMA: Preferred Reporting Items for Systematic Reviews and Meta-Analyses

A total of 66 studies were identified through database search, and their titles and abstracts were reviewed. Forty-three studies were deemed eligible for full-text review. Two studies were excluded because their full texts were not in English. Eight studies were excluded due to unrelated content (Figure 3).

Finally, 33 studies (36 individual cases including 24 males and a mean age of 54.4 ± 13.9 years) were identified, including the present case [7-39]. Their data are presented in Table 1. Alcoholism was the most common comorbidity (n = 13; 36.1%). The median delay in the final diagnosis was four days. Of these 36 patients, 18 (50.0%) developed congestive heart failure, and 10 (27.8%) developed a brain abscess. Valve vegetation was observed at the aortic valve in 17 (47.2%) patients, at the mitral valve in 13 (36.1%), and at both the aortic and mitral valves in five (13.9%) patients. Ceftriaxone and vancomycin were the most commonly administered antibiotics. Valve replacement surgery was performed in 22 (61.1%) patients. Eleven (30.6%) patients died.

**Table 1:** Summary of literature search ND, No Data; AC, acromioclavicular; Af, atrial fibrillation; AKI, acute kidney injury; ARDS, acute respiratory distress syndrome; CAD, coronary artery disease; CHF, congestive heart failure; CPA, cardiorespiratory arrest; CVD, cerebrovascular disease; DRESS, Drug reaction with eosinophilia and systemic symptom; Dx, diagnosis; HT, hypertension; ITP, Immune thrombocytopenic purpura; PAD, peripheral artery disease; Paf, paroxysmal atrial fibrillation; PE, Pulmonary embolism; RA, Rheumatoid Arthritis; T2DM, Type 2 diabetes mellitus; TEN, toxic epidermal necrolysis; ACY, acyclovir; AMP, ampicillin; AMX, amoxicillin; AZM, azithromycin; CEP, cephalosporin; CFT, cefotaxime; CTM, cefotiam; CTX, ceftriaxone; GM, gentamicin; LVFX, levofloxacin; MEPM, meropenem; MFLX, moxifloxacin; PIPC/TAZ, piperacillin/tazobactam; RFP, rifampicin; VCM, vancomycin

No.	Cases	Age (yrs)	Sex	Delay (in days)	Comorbidities	Cerebral infarction	Dx in CI (day)	GCS on admission	Cardiac complication	Valve involved	Other complication	Initial antiboitic therapy	Valve replaced	Outcome
1	Akram et al., 2020 [7]	67	M	ND	None	Multiple	1	confusion	None	Mitral	General seizure	ND	None	Survived
2	Bakhit et al., 2021 [8]	52	M	ND	None	Multiple	ND	-	CHF	Aortic	None	CTX, VCM	Aortic	Survived
3	Belvisi et al., 2013 [9]	61	F	ND	Splenectomy, liver transplantation	None	-	12	None	Mitral	Brain abscess, spondylodiscitis	CFT	None	Survived
4	Bindroo et al., 2018 [10]	60	M	ND	Bicuspid aortic valve	Multiple	1	8	None	Aortic	Right shoulder septic arthritis	CTX	Aortic	Survived
5	Chiu et al., 2020 [12]	63	F	ND	HIV	ND	-	coma	None	Mitral	Septic shock, DRESS, TEN	CTX	None	Died
6	Dalal et al., 2008 [13]	55	M	1	Alcoholism, CAD, PAD	ND	-	8	CHF	Aortic	MOF	CTX, VCM	Aortic	Survived
7	du Cheyron et al., 2003 [14]	51	M	1	PAD, HT	None	-	8	CHF	Aortic	Septic shock, MOF, aortic abscess	CFT, VCM, AMX	Aortic	Survived
8		70	F	2	None	None	-	7	None	Mitral	Septic shock, AKI	CFT, VCM	None	Survived
9	Edlin et al., 2020 [15]	43	M	8	Reccurent gallstone pancreatitis, bicuspid aortic valve	Multiple	-	confusion	CHF	Aortic, Mitral	Septic shock, aortic root abscess, unheralded CPA	CTX, ACY	Aortic, Mitral	Survived
10	Favre et al., 2003 [16]	50	M	ND	Alcoholism, T2DM	None	-	confusion	CHF	Aortic	Brain abscess	CFT, RFP	Aortic	Survived
11	Georgiadou et al., 2018 [17]	52	M	ND	Alcoholism, illegal drug abuse	ND	-	-	CHF	Aortic	ARDS	CTX, VCM, AMP	Aortic	Survived
12	Gonzalez-Juanatey et al., 2006 [18]	45	M	7	Alcoholism	ND	-	coma	CHF	Aortic	None	VCM	Aortic	Survived
13		61	F	2	None	ND	-	confusion	None	Mitral	Septic shock, brain abscess	CFT	None	Died
14	Guerreiro et al., 2022 [19]	84	F	4	ITP, RA	Multiple	2	14	None	Mitral	Septic shock	CTX, VCM	None	Died
15	Kanakadandi et al., 2013 [20]	61	M		Bicuspid aortic valve, HT, T2DM	None	-	4	CHF	Aortic	Empyema	CTX, VCM, AZM	Aortic	Survived
16	Kim et al., 2011 [21]	59	M	5	None	Multiple	5	-	CHF	Aortic	None	CEP	Aortic	Survived
17	Luzzati et al., 2007 [22]	39	F	5	Hodgkin's lymphoma, splenectomy	None	-	confusion	CHF	Aortic	Aortic right atrial fistula	CFT, AMP	Aortic	Survived
18	Mankongpaisarnrung et al., 2012 [23]	52	M	ND	Alcoholism, splenectomy, Paf	None	-	7	None	Aortic, Mitral	Right renal infaction	PIPC/TAZ, LVFX, VCM	Aortic, Mitral	Died
19	Mekraksakit et al., 2020 [25]	60	M	3	Illegal drug abuse	None	-	coma	None	Tricuspid	PE, psoas abscess	CTX, VCM	Tricuspid	Survived
20	Midon et al., 2011 [26]	64	M	ND	None	rt.hemisphere	ND	confusion	None	Mitral	ARDS	CTX, VCM	None	Survived
21	Miyaue et al., 2020 [27]	59	M	2	Alcoholism	lt.hemisphere	28	-	None	Mitral	Septic arthritis of the AC joint	CTM	Mitral	Survived
22	MuCulloch et al., 2021 [24]	75	F	4	COPD	None	-	11	CHF	Mitral	AKI	CTX, VCM, ACY	None	Died
23	Pandey et al. 2022 [28]	58	M	ND	Alcoholism, CVD	None	-	7	CHF	Aortic	Spetic shock, ARDS, Af	CTX, VCM, AMP, ACY	Aortic	Survived
24	Pawar et al., 2017 [29]	41	M	ND	Autism	Multiple	15	11	None	Aortic	Multidrug-resistant Acinetobacter baumannii pneumoniae	PIPC/TAZ	None	Died
25	Poulsen et al., 2011 [30]	49	F	5	Alcoholism	None	-	9	CHF	Aortic	Septic shock	CTX, AMP	Aortic	Survived
26	Rahim et al., 2017 [31]	48	M	4	T2DM	None	-	11	None	Mitral	None	CTX, MFLX	None	Survived
27	Rakočević et al., 2019 [32]	76	F	17	HT, T2DM	Multiple	17	9	None	Aortic	ARDS	CTX, VCM, AMP, ACY	None	Died
28	Rammeloo et al., 2004 [33]	7	F	ND	None	None	-	-	None	Aortic	None	CTX, VCM	Aortic	Survived
29	Shin et al., 2020 [34]	43	M	2	Alcoholism	ND	-	confusion	CHF	Aortic	Septic shock, DIC	CTX, VCM	Aortic	Died
30	Shokoohi et al., 2015 [35]	30	F	1	Illegal drug abuse	rt.hemisphere	1	15	CHF	Aortic, Mitral	ARDS	CTX, VCM	Aortic, Mitral	Survived
31	Velazquez et al., 2008 [36]	55	M	10	Alcoholism	ND	-	confusion	CHF	Aortic	None	CTX	Aortic	Survived
32	Vindas-Cordero et al., 2009 [37]	49	F	ND	Alcoholism	ND	-	-	None	Mitral	Cardiac tamponade, left shoulder septic arthritis	CTX, VCM, GM	None	Survived
33	Watchmaker et al., 2021 [38]	58	M	23	Alcoholism, HCV carrier, illegal drug abuse	None	-	-	None	Mitral	ARDS, septic shock	CTX, VCM, PIPC/TAZ	Aortic, Mitral	Survived
34	Wilbring et al., 2012 [39]	41	M	ND	Alcoholism	None	-	-	CHF	Aortic, Mitral	ARDS, left atrium fistula	ND	Aortic, Mitral	Died
35	Zheng et al., 2018 [11]	54	M	4	None	Multiple	4	15	CHF	Aortic, Mitral	General seizure, AKI	CTX	None	Died
36	Present case	68	M	1	None	Multiple	1	12	None	Mitral	AKI, unheralded CPA	VCM, MEPM	None	Died

Table 2 shows the results of the univariate and multivariate analyses for each factor. Unfavorable outcomes were significantly associated with surgery (p=0.023). Austrian syndrome initiated by cerebral infarction was not associated with unfavorable outcomes.

**Table 2:** The risk factors of unfavorable outcomes Values are n (%) except for age, which is in mean±SD ARDS, acute respiratory distress syndrome; CHF, congestive heart failure; GCS, Glasgow coma scale; CTX, ceftriaxone; VCM, vancomycin; OR, odds ratio; SD, standard deviation

				Univariate analysis		Multivariate analysis	
		Survived (n=25), n (%)	Died (n=11), n (%)	OR (95%CI)	p-value	OR (95%CI)	p-value
Patients characteristics	Age (years), mean ± SD	52.1 ± 12.7	59.8 ± 14.3		0.204	0.95（0.89-1.03）	0.20
	Sex (male)	18 (72.0)	6 (54.5)	2.14 (0.49-9.35)	0.446	2.08(0.25-17.52)	0.50
	Alcoholism	10 (40.0)	3 (27.3)	1.78 (0.38-8.37)	0.708	0.37(0.035-3.90)	0.41
	Immunodeficiency	2 (8.0)	2 (18.2)	0.39 (0.05-3.21)	0.571	3.84(0.21-20.23)	1.00
Clinical complications	Cerebral infarction on admission	3 (12.0)	2 (18.2)	0.61 (0.09-4.31)	0.631	0.33(0.04-3.10)	0.33
	Cerebral infarction in hospitalization	8 (32.0)	5 (45.5)	0.56 (0.13-2.42)	0.475	0.33(0.03-3.22)	0.34
	GCS on admission, median	8	11		0.134		
	CHF	14 (56.0)	4 (36.4)	2.23 (0.52-9.59)	0.471	0.37(0.04-3.39)	0.38
	Valve involved						
	Aortic	14 (56.0)	3 (27.3)	3.39 (0.72-15.90)	0.156	0.44(0.02-3.32)	0.43
	Mitral	8 (32.0)	5 (45.5)	0.56 (0.13-2.42)	0.475	0.65(0.03-4.07)	0.23
	Aortic & Mitral	2 (8.0)	3 (27.3)	0.23 (0.03-1.65)	0.154	0.1(0.09-1.56)	0.54
	Tricuspid	1 (4.0)	0	1.41 (0.05-37.29)	1	0.41(0.03-5.33)	0.44
	Septic shock	6 (24.0)	4 (36.4)	0.55 (0.12-2.56)	0.454	4.48(0.38-52.93)	0.23
	ARDS	5 (20.0)	2 (18.2)	1.13 (0.18-6.94)	1	1.10(0.09-13.66)	0.94
	Any abscess	9 (36.0)	1 (9.1)	5.63 (0.62-51.38)	0.127	4.96(0.10-244.04)	0.42
Treatment	CTX and/or VCM use	19 (76.0)	8 (72.7)	1.19 (0.24-5.96)	1	0.41(0.04-4.33)	0.46
	Valve replacement	19 (76.0)	3 (27.3)	8.44 (1.68-42.39)	0.001	9.00 (1.36-59.78)	0.023

Discussion

Austrian syndrome has been seen more commonly in middle-aged, debilitated men with a history of chronic alcoholism [13,40]. Other risk factors for invasive pneumococcal infections include higher age, diabetes mellitus, chronic renal insufficiency, chronic liver disease, chronic pulmonary disease, anatomical or functional asplenia, tobacco abuse, and other immunosuppressive conditions such as human immunodeficiency virus and multiple myeloma [41]. In the present case, the patient's medical history was unclear, and these predisposing conditions could have existed without our knowledge.

The diagnosis of IE is usually delayed due to the late occurrence of cardiac murmur and other classical signs of endocarditis [14,42]. Early detection of cardiac involvement, especially in Austrian syndrome, is difficult because the initial symptoms are often explained by pneumonia or central nervous system involvement [43,44].

The clinical course of pneumococcal endocarditis is usually acute and very aggressive, with a high rate of mortality (non-surgical: 60%, early surgery: 32%) and association with the rupture of the aortic valve [2,3,36]. In most cases of Austrian syndrome, despite adequate antibiotic therapy, pneumococcal endocarditis was acutely progressed, and the median time of diagnosis was one to seven days after antibiotic therapy of bacterial meningitis with a newly developed dyspnea and/or cardiac murmur by valve destruction [13,14,18,33,36]. Subacute evolution is less frequent and often involves mitral endocarditis [45]. In our case, the vegetation was detected at the mitral valve, but the clinical progression was drastic.

Patients with IE are prone to hemorrhagic and embolic events [8] Septic cerebral embolism has been reported in approximately 40% of patients with IE [8]. IE involving the left-sided valve is prone to cerebral embolization [22]. Risk factors for developing embolic infarcts are advanced age, history of embolic events, multiple valvular endarterectomies, more extensive vegetation, and pathogenic organisms such as *Staphylococcus aureus* and *Candida* [46]. Reports of pneumococcal embolic events are uncommon. These embolic infarcts are multiple, often bilateral, and tend to be distributed in the middle cerebral artery territory [47]. The onset of embolism occurs most frequently before antimicrobial therapy, and appropriate antimicrobial therapy reduces the risk of embolic complications [48-54]. Therefore, rapid diagnosis and then immediate appropriate antimicrobial therapy are most critical to prevent embolism. The incidence of embolism after antimicrobial therapy is high in the first few days after initiation of therapy and then rapidly declines, rarely occurring more than two weeks after initiation [54].

Early surgical therapy should be considered in cases of advanced heart failure, rupture of intracardiac structures, refractory infection, and possible embolism [55]. Although there have been negative opinions about early surgery for patients with concomitant cerebral infarction, it has recently become clear that the prognosis is not as bad as previously thought when early surgery is performed for patients with concomitant cerebral infarction [55-60]. Barsic et al. reported in a multicenter prospective registry study that neither in-hospital mortality nor one-year mortality was significantly worse when early surgery was performed within seven days of stroke onset compared with surgery performed after seven days [61]. Although surgical mortality was higher for cerebral infarction, cerebral hemorrhage, meningitis, and brain abscess in the middle cerebral artery territory, there was no significant difference in the incidence of perioperative central nervous system complications or mortality in early cardiac surgery compared to patients without cerebral infarction [60].

Our literature review also indicated no difference in mortality with or without ischemic stroke. Hence, it is essential to check the patient's condition frequently to ensure that the appropriate time for surgery is not missed.

In the present case, the patient experienced cardiopulmonary arrest owing to cardiogenic shock after hospitalization. The patient's consciousness did not improve due to hypoxic encephalopathy. Since multiple cerebral infarcts were observed, cardiogenic embolization should have been suspected, and transthoracic echocardiogram and TEE should have been performed sooner to identify verrucous lesions. Early detection of verrucous verrucae and the suspicion of cardiogenic embolization in cases of multiple cerebral infarctions with fever may have allowed the patient to undergo valve replacement surgery before circulatory failure.

## Conclusions

In the case discussed in this report, Austrian syndrome initially presented as cerebral infarction, but no association with mortality was found. Valve vegetation results in congestive heart failure and this might have been related to patient mortality; therefore, valve replacement surgery at an adequate period is critical for patient mortality regardless of the presence or absence of cerebral infarction.
